# Fine morphological assessment of quality of human mature oocytes after slow freezing or vitrification with a closed device: a comparative analysis

**DOI:** 10.1186/1477-7827-12-110

**Published:** 2014-11-24

**Authors:** Veronica Bianchi, Guido Macchiarelli, Andrea Borini, Michela Lappi, Sandra Cecconi, Selenia Miglietta, Giuseppe Familiari, Stefania A Nottola

**Affiliations:** Casa di Cura Città di Udine, Udine, Italy, affiliated to Tecnobios Procreazione, Centre for Reproductive Health, Bologna, Italy; Department of Life, Health and Environmental Sciences, University of L´Aquila, L’Aquila, Italy; Department of Anatomy, Histology, Forensic Medicine and Orthopaedics, La Sapienza University, Rome, Italy

**Keywords:** Oocyte, Cryopreservation, Slow freezing, Vitrification, Ultrastructure, Human

## Abstract

**Background:**

Human mature oocytes are very susceptible to cryodamage. Several reports demonstrated that vitrification might preserve oocyte better than slow freezing. However, this is still controversial. Thus, larger clinical, biological and experimental trials to confirm this concept are necessary. The aim of the study was to evaluate and compare fine morphological features in human mature oocytes cryopreserved with either slow freezing or vitrification.

**Methods:**

We used 47 supernumerary human mature (metaphase II) oocytes donated by consenting patients, aged 27-32 years, enrolled in an IVF program. Thirtyfive oocytes were cryopreserved using slow freezing with 1.5 M propanediol +0.2 M sucrose concentration (20 oocytes) or a closed vitrification system (CryoTip Irvine Scientific CA) (15 oocytes). Twelve fresh oocytes were used as controls. All samples were prepared for light and transmission electron microscopy evaluation.

**Results:**

Control, slow frozen/thawed and vitrified/warmed oocytes (CO, SFO and VO, respectively) were rounded, 90–100 μm in diameter, with normal ooplasm showing uniform distribution of organelles. Mitochondria-smooth endoplasmic reticulum (M-SER) aggregates and small mitochondria-vesicle (MV) complexes were the most numerous structures found in all CO, SFO and VO cultured for 3–4 hours. M-SER aggregates decreased, and large MV complexes increased in those SFO and VO maintained in culture for a prolonged period of time (8–9 hours). A slight to moderate vacuolization was present in the cytoplasm of SFO. Only a slight vacuolization was present in VO, whereas vacuoles were almost completely absent in CO. Amount and density of cortical granules (CG) appeared abnormally reduced in SFO and VO, irrespective of the protocol applied.

**Conclusions:**

Even though, both slow freezing and vitrification ensured a good overall preservation of the oocyte, we found that: 1) prolonged culture activates an intracellular membrane “recycling” that causes the abnormal transformation of the membranes of the small MV complexes and of SER into larger rounded vesicles; 2) vacuolization appears as a recurrent form of cell damage during slow freezing and, at a lesser extent, during vitrification using a closed device; 3) premature CG exocytosis was present in both SFO and VO and may cause zona pellucida hardening.

## Background

Thanks to the important technological advances obtained in the last two decades, human oocyte cryopreservation is today one of the techniques of choice in clinical practice. In respect to embryo cryopreservation, oocyte cryopreservation may be applicable even in absence of a male partner. In addition, oocyte freezing may circumvent ethical or legal considerations associated with embryos [1]. However, although the first human live birth from cryopreserved oocytes was reported more than twenty years ago [2], nevertheless the success rates in assisted reproductive technologies using frozen oocytes have lagged behind those using frozen embryos, most likely as a result of the biochemical and physical properties of the oocyte.

Cell survival after freezing is intimately associated with the composition and the permeability characteristics of the cell membrane, the surface to volume ratio of the cells, and the difference in osmotic pressure between the two sides of the membranes [3, 4]. Additional factors associated with oocyte survival and developmental competence after freezing include developmental stage at freezing (isolation at the germinal vesicle – GV -, meiotic metaphase I, vs. meiotic metaphase II – MII - stages of development), cryoprotectant type and concentration and, the method or “protocol” of cryopreservation. Oocyte survival with a specific protocol can also vary according to species. This is mainly related to the oocyte size, but also biochemical properties contribute to such an outcome. Mature MII oocytes, commonly used for cryopreservation, are among the largest cells in the human body and contain the delicate meiotic spindle. As their cytoplasm contains a high proportion of water in comparison to other cells, damage due to ice crystal formation was a mandatory problem to overcome to achieve viability after thawing [5, 6]. Protocols that include dehydration of oocytes before and/or during the cooling procedure reduced ice crystal formation and improved clinical outcomes [7]. Cryopreservation of mature oocytes may cause hardening of the zona pellucida (ZP), impairing fertilization [8]; however, intracytoplasmic sperm injection (ICSI) may be applied for overcoming this problem [9].

Up to date, the two most common freezing protocols used are slow freezing and vitrification [10]. In slow freezing protocol, the oocytes are gradually frozen (controlled rate freezing) in the presence of low concentrations of cryoprotectants, which reduce the risk of intracellular ice formation. On the contrary, the vitrification method combines ultrarapid cooling with minimum volume and high concentration of cryoprotectants, avoiding the formation of ice crystals, and thus giving to the vitrification solution a transparent, glass-like appearance [7].

In the last years, each protocol showed outcome improvement [11, 12], although cell damage resulted lower after vitrification in some reports [13, 14]. Indeed, larger controlled clinical, biological and experimental trials are necessary to eventually confirming these data. In particular, it is still controversial the opportunity of using open devices versus closed devices for vitrification [15–18]. Open devices seem superior to closed ones, at least according to the little morphological data available [19].

Evaluation of oocyte quality after cryopreservation is based mainly on the morphological appearance of the oocyte [20]. Phase contrast microscopy (PCM) is currently used for scoring oocyte and embryo quality. However, a good survival as evaluated by standard PCM is not necessary related to a good performance of the cell in giving a competent embryo. At this extent, the use of electron microscopy (EM) to evaluate fine morphological damage is a tool to assess oocyte quality to a higher sensible level. Transmission electron microscopy (TEM), especially when associated with a morphometric analysis, allows an accurate evaluation of fine details of cell microanatomy that can be compromised during the cryopreservation procedures [21–27].

The aim of this study was to compare the effects of slow freezing and vitrification with a closed device on the quality of the oocyte. In this article, we report correlated light microscopy (LM) and TEM observations and morphometric data on the fine morphology of human fresh and cryopreserved MII oocytes.

## Methods

### Source of oocytes

This project has been approved by our internal review board and the Italian Ministry of Health. The study involves patients who decided to donate their supernumerary eggs to research between January 2012 and January 2013. The age of women ranged from 27 to 32 years old, and their infertility was due to male, tubal factors or idiopathic issues. Patients with endometriosis or other conditions, that could influence oocyte quality, were excluded from the study. Ovarian stimulation was induced with a long protocol using GnRH agonist and rFSH, according to previous clinical report [28]. HCG (10,000 IU) was injected 36 hours before retrieval. Oocytes were cultured in fertilization media for at least 2 hours (Cook IVF, Brisbane, Australia) before cumulus/corona cells complete removal; this was performed enzymatically (hyaluronidase 20–40 IU/ml) and mechanically (using fine pipettes Flexi pipette Cook). Mature oocytes displaying a 1st polar body (PB) with a clear cytoplasm were assigned to either the fresh control or study groups. Fresh control oocytes (CO) were fixed after a period of 3–4 hours following retrieval. Cryopreservation was performed within 3–8 hours after retrieval according to the laboratory workload. Slow frozen/thawed (SFO) and vitrified/warmed oocytes (VO) were cultured for a further hour before fixation.

### Freeze-thawing procedure

Oocytes were cryopreserved using a slow-freezing protocol adapted from the one originally described for embryos [29]. Eggs were equilibrated in one step homemade solution containing 1.5 mol/L 1,2-propanediol (PrOH) supplemented with 20% of synthetic serum supplement (SSS) (Irvine Scientific, Santa Ana, CA, USA) for 10 minutes then transferred for 5 minutes into a loading solution containing 1.5 mol/L PrOH +0.2 mol/L sucrose +20% SSS. Afterwards the oocytes were loaded into plastic straws (Paillettes Crystal 133 mm; CryoBioSystem, Paris, France) and placed into an automated Kryo 10 series III biological freezer (Planer Kryo 10/1,7 GB; Planer, Surrey, UK). All the procedures were carried out at room temperature (around 25°C). Once the loaded straws were placed in the machine the temperature was gradually lowered from 20°C to -7°C at a rate of -2° C/min. Manual seeding was induced during a 10 minutes holding ramp at -7°C. The temperature was then decreased to -30° C at a rate of -0.3°C/min and finally rapidly to -150° C at a rate of -50°C/min. The straws were then plunged into liquid nitrogen and stored for later use. For the thawing procedures the straws were air-warmed for 30 seconds and then placed in a 30°C water bath for 40 seconds. The cryoprotectant was removed by stepwise dilution of PrOH at room temperature. The thawing solutions were homemade and contained:

- 1.0 mol/L PrOH +0.3 mol/L sucrose +20% SSS (5 minute equilibration)
- 0.5 mol/L PrOH +0.3 mol/L sucrose +20% SSS (5 minute equilibration)
- 0.3 mol/L sucrose +20% SSS (10 minute exposure before the final dilution in PBS solution for another 10 minutes).

Survived oocytes were finally placed in culture in Cleavage medium (Cook) at 37°C and 5% CO_2_ and re-checked 1 hour later to confirm their good status prior to fixation.

### Vitrification-warming procedure

Oocytes were vitrified using commercial kits (Irvine Scientific CA) and closed vitrification devices (CryoTip). The oocytes were washed in a drop of hepes-buffered medium (gamete buffer Cook) and, subsequently in three drops of equilibration solution containing ethylene glycol (EG) (7.5% vol/vol) and dimethylsulphoxide (DMSO) (7.5% vol/vol). After 8 minutes, eggs were transferred into a vitrification solution drop containing 15% vol/vol EG, 15% vol/vol DMSO, and 0.5 mol/L sucrose, for a total of 20 seconds before being loaded into a Cryotip and sealed properly at both ends. The device was directly plunged into liquid nitrogen and stored.

Oocytes were rapidly warmed by transferring the Cryotip directly from liquid nitrogen to a 37°C water bath for 3 seconds. The device was cut at the end and the oocyte/s released in a thawing solution (1.0 mol/L sucrose) for a minute then moved to a dilution media (0.5 mol/L sucrose) for 4 minutes and finally washed twice in a washing solution (6 minutes). Survived oocytes were cultured in Cleavage medium (Cook) before the fixation.

### Electron microscopy

Fortyseven supernumerary human MII oocytes (12 CO, 20 SFO and 15 VO) were donated by consenting patients (N = 12), and included in this study. In detail: 3 patients donated 12 CO (4 oocytes from each patient), 5 patients donated 20 SFO (2, 3, 4, 5, 6 oocytes respectively from each patient), 4 patients donated 15 VO (1, 3, 6, 5 oocytes respectively from each patient).

Only oocytes that appeared of good quality when observed by PCM after thawing or warming were selected for EM evaluation. They should have: 1. a rounded, regular shape; 2. a clear, moderately granular cytoplasm; 3. a narrow perivitelline space (PVS) with the 1st PB and, 4. an intact, colorless ZP [30].

Oocytes were fixed and processed for LM and TEM analysis as follows. Oocyte fixation was performed in 1.5% glutaraldehyde (SIC, Rome, Italy) in PBS solution. After fixation for 2–5 days at 4°C, the samples were rinsed in PBS, post-fixed with 1% osmium tetroxide (Agar Scientific, Stansted, UK) in PBS, and rinsed again in PBS. Oocytes were then embedded in small blocks of 1% agar of about 5×5×1 mm in size, dehydrated in ascending series of ethanol (Carlo Erba Reagenti, Milan, Italy), immersed in propylene oxide (BDH Italia, Milan, Italy) for solvent substitution, embedded in epoxy resin (Electron Microscopy Sciences, Hatfield, PA, USA) and sectioned by a Reichert-Jung Ultracut E ultramicrotome. Semithin sections (1 μm thick) were stained with toluidine blue, examined by LM (Zeiss Axioskop) and photographed using a digital camera (Leica DFC230). Ultrathin sections (60–80 nm) were cut with a diamond knife, mounted on copper grids and contrasted with saturated uranyl acetate followed by lead citrate (SIC, Rome, Italy). They were examined and photographed using a Zeiss EM 10 and a Philips TEM CM100 Electron Microscopes operating at 80KV. Images were acquired using a GATAN CCD camera.

According to Nottola et al. [23], as reviewed by Khalili et al. [26], the following parameters have been evaluated by LM and TEM and taken into consideration for the qualitative morphological assessment of the ultrastructural preservation of oocytes: main characteristics (including shape and dimensions), ZP texture, PVS appearance, integrity of the oolemma, microtopography, type and quality of the organelles, presence and extent of ooplasmic vacuolization.

The presence and characteristics of the 1st PB in the PVS and the arrangement of the MII spindle were not systematically assessed, due to their detection only in sections laying on appropriate planes.

### Statistical analysis

The presence of vacuoles ≥1 μm was evaluated at the LM level on at least 3 equatorial sections per oocyte (distance between the sections: 3–4 μm), and values were expressed in number of vacuoles per 100 μm^2^ of the oocyte area. The evaluation of cortical granule (CG) density was performed through collection of TEM microphotographs of the entire surface profiles at a magnification of 6300X on 3 equatorial sections per oocyte. Images were further enlarged on the PC screen, in order to easily recognize and count CG. Values were expressed in number of CG per 10 μm of the oocyte linear surface profile [23].

Authors presented statistical data as a mean value ± standard deviation (SD); P value and significance were evaluated using Student’s t test (http://www.graphpad.com/quickcalcs, last access: September 2, 2014). The cut off for significance was P < 0.05.

## Results

### Main characteristics

**Figure 1:**
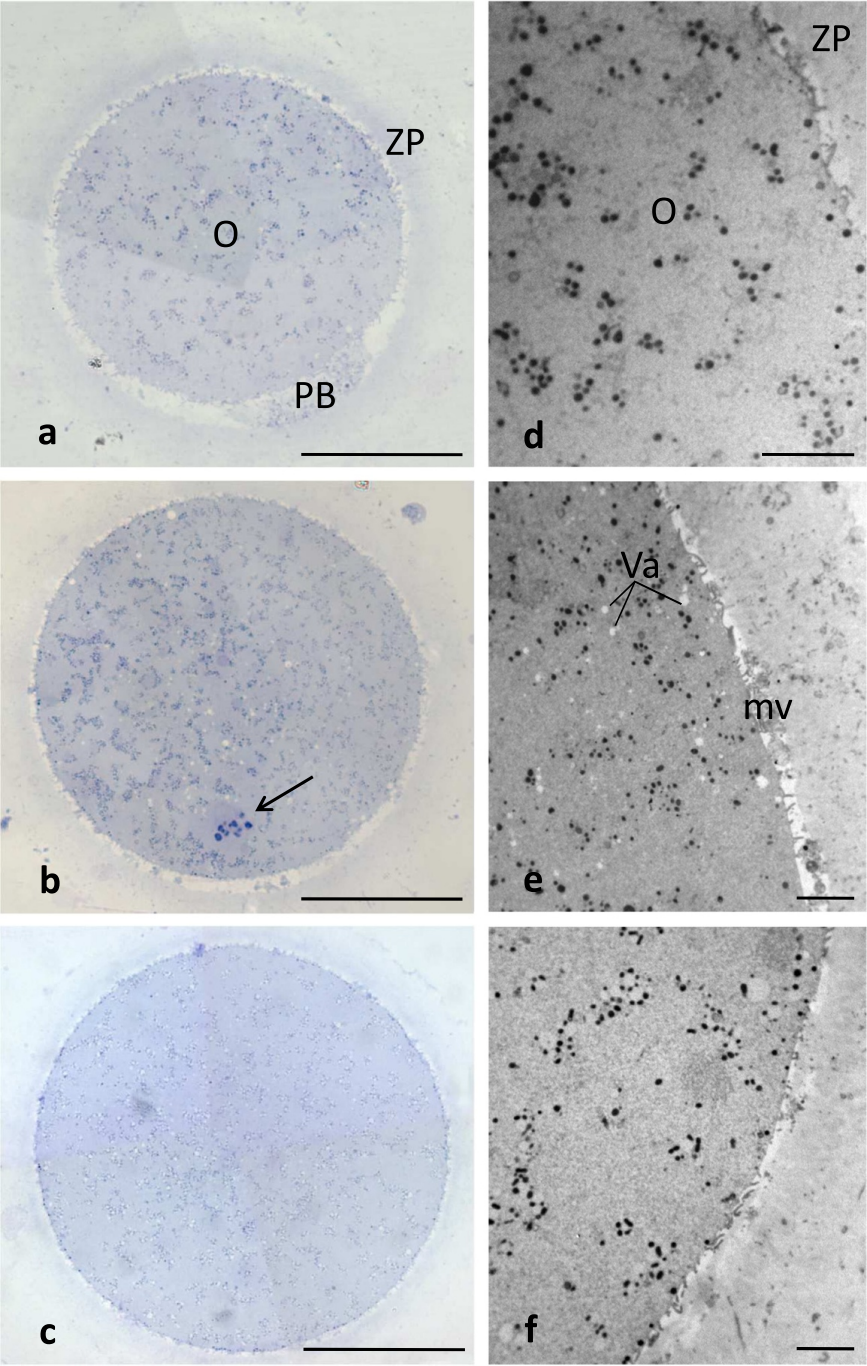
**Human CO, SFO and VO at MII stage.** Main characteristics. By LM **(a-c)** and TEM **(d-f)** no overt difference in shape, dimensions and organelle distribution is seen among CO **(a,d)**, SFO **(b,e)** and VO **(c,f)**. Note the intact ZP **(a-c)** and the presence of microvilli (mv) on the oolemma **(d-f)**. Numerous vacuoles (Va) are seen in SFO **(b,e)**. O: oocyte; PB: 1st PB; arrow: MII spindle with chromosomes. Bar is: 45 μm **(a-c)**; 5 μm **(d-f)**.

LM and TEM techniques allowed analyzing and comparing size, shape and organelle distribution in CO, SFO and VO. CO, SFO and VO were all generally rounded, 90–100 μm in diameter, with normal ooplasm showing uniform distribution of organelles. All CO, SFO and VO showed an intact ZP, separated by a narrow PVS from the oolemma, continuous and provided with microvilli (Figure 1a, b, c, d, e and f).

Only in favorable sections, the 1st PB was detected by LM in the PVS (which appeared wider in this region) (Figure 1a). The MII spindle (or a portion of this) was also visible in the ooplasm of a number of CO (N = 4), SFO (N = 6) and VO (N = 5), assuming a peripheral position (Figure 1b). By TEM, the 1st PB contained condensed chromatin, mitochondria, residual microtubules and scattered CG. The MII spindle consisted of chromosomes, with a dense granulo-fibrillar microstructure, and associated microtubules converging at each pole (data not shown).

### Mitochondria-smooth endoplasmic reticulum aggregates and mitochondria-vesicle complexes

**Figure 2:**
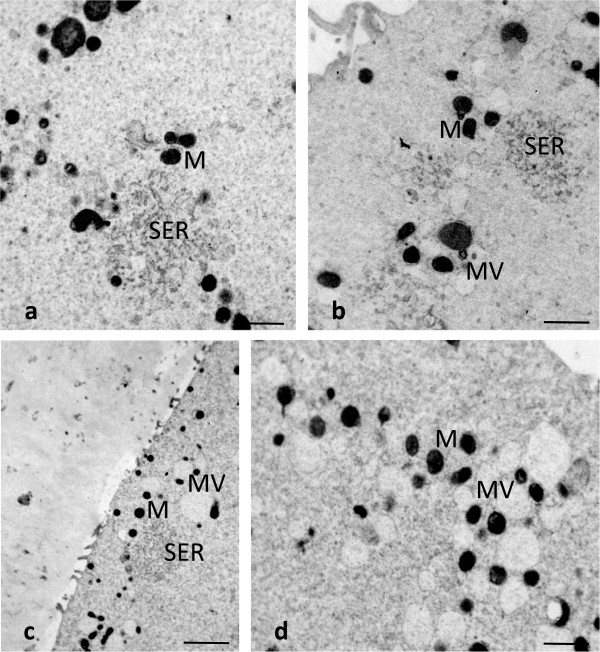
**Human CO, SFO and VO at MII stage.** M-SER aggregates and MV complexes. By TEM, voluminous M-SER aggregates are seen in **(a)** (CO) and **(b)** (SFO cultured for 3–4 hours). Small MV complexes are also observable in **(b)**. M-SER appear partially replaced by numerous and larger MV complexes after prolonged culture, as seen in **(c,d)** (VO cultured for 8–9 hours). Mitochondria (M) are well preserved in both SFO **(b)** and VO **(d)**. Bar is: 1 μm **(a,b,d)**; 2 μm **(c)**.

**Figure 3:**
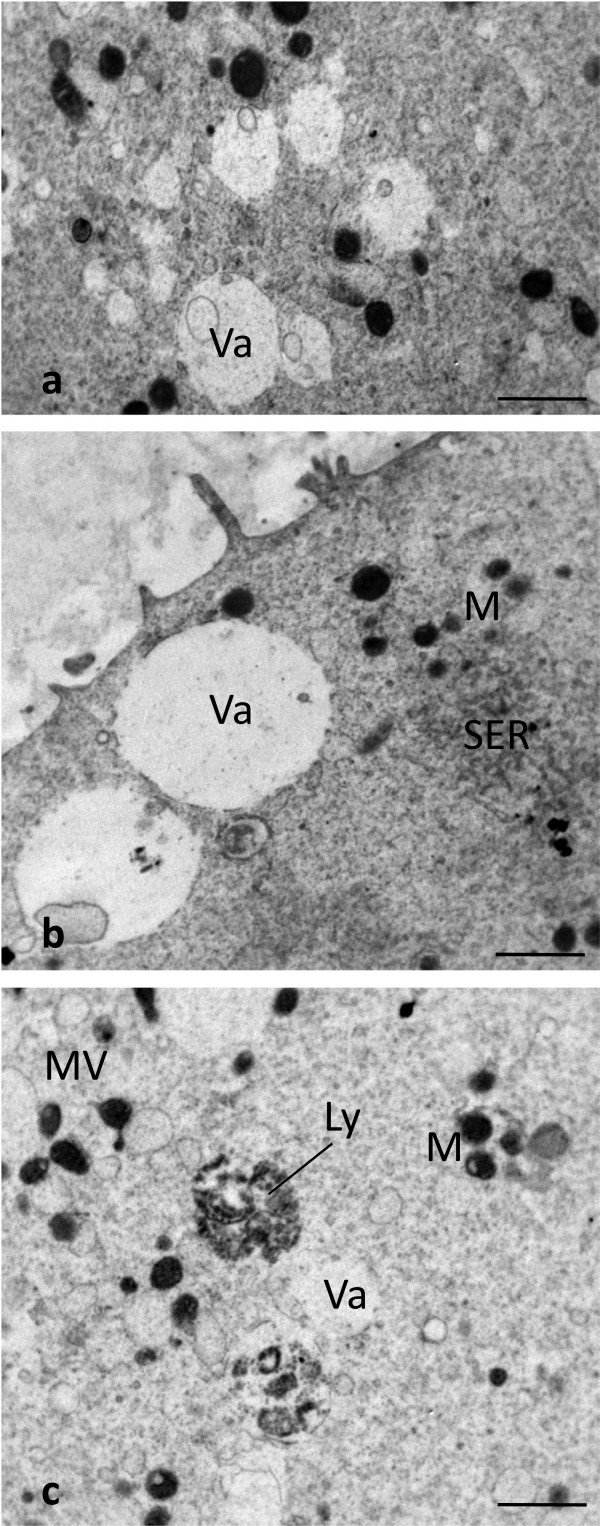
**Human SFO and VO at MII stage.** Presence of vacuoles. By TEM, numerous vacuoles (Va) are seen in the ooplasm of SFO **(a,b)**, whereas only a few vacuoles are visible in VO **(c)**. Vacuoles appear empty **(a-c)** or contain cell debris **(a,b)**. Note some interruptions in the vacuole membrane **(a-c)**. A close association between a vacuole and a lysosome (Ly) is seen in **(c)**. A typical M-SER aggregate **(b)**, isolated mitochondria (M) and MV complexes **(c)** are seen in the areas adjacent to vacuoles. Bar is: 1 μm **(a-c)**.

Using TEM, the most numerous organelles found in all the CO, SFO and VO cultured for 3–4 hours, consisted of aggregates of anastomosing tubuli of smooth endoplasmic reticulum (SER) surrounded by mitochondria (M-SER aggregates). The diameter of the tubular network of the M-SER aggregates varied from 1 to 5 μm (Figure 2a and b). Small vesicles, 0.3 – 0.5 μm in diameter, containing a slighlty electrondense material were associated with mitochondria forming the so-called mitochondria-vesicle (MV) complexes (Figure 2b). No overt qualitative differences in the fine structural morphology of M-SER aggregates and MV complexes came out from the comparison among CO, SFO and VO after 3–4 hours of culture. M-SER aggregates appeared instead partially replaced by numerous, large MV complexes, up to 2.5 μm in vesicle diameter, when SFO and VO extended culture for a prolonged period of time (8–9 hours) (Figure 2c and d). Mitochondria, either associated with membranes or isolated, revealed a normal fine structure in all the samples observed (CO, SFO and VO). They were rounded or oval in profile, with a diameter varying from 0.5 to 0.8 μm and a few peripheral arch-like or transverse cristae and contained a moderately electrondense matrix (Figure 2a, b and d; Figure 3c).

### Vacuoles

LM showed very rare vacuolization of the ooplasm in CO (Figure 1a). TEM confirmed the very sporadic incidence of vacuolization in these oocytes (Figure 1d). A slight to moderate vacuolization was found in the cytoplasm of cryopreserved oocytes, more pronounced in SFO than in VO (Figure 1b and e; Figure 3a, b and c). Vacuoles, varying in diameter from 0.2 to about 2.5 μm, were apparently empty (Figure 3a,b and c) or contained cell debris (Figure 3a and b). Vacuole membrane was often discontinuous (Figure 3a, b and c). Sometimes, secondary lysosomes and/or multivesicular bodies (MVBs) associated with vacuoles were present (Figure 3c). Otherwise, it was usually detected a normal pattern of organelles in the cytoplasmic areas adjacent to vacuoles (Figure 3b and c).

The morphometric analysis performed on fresh and cryopreserved oocytes belonging to both study groups (i.e. subjected to slow freezing or vitrification with a closed system) revealed that the difference in vacuole number between CO and SFO was statistically significant. In particular, the mean number ± SD of vacuoles ≥1 μm in diameter per 100 μm^2^ was 0.40 ± 0.12 and 2.09 ± 0.85 in CO and SFO, respectively (P < 0.0001) (Table 1). A statistically significant difference in vacuole number between CO and VO was also present. In fact, the mean number ± SD of vacuoles ≥1 μm in diameter per 100 μm^2^ was 0.40 ± 0.12 and 0.85 ± 0.37 in CO and VO, respectively (P = 0.0005) (Table 1). However, we found a significant difference in vacuole number also between SFO and VO (P < 0.0001), confirming the presence of more vacuoles in SFO than in VO (Table 1).

**Table 1:** **Morphometric evaluation of the presence and extent of vacuolization in human MII oocytes subjected to slow freezing and vitrification with a closed device**

	Fresh controls (CO)	Cryopreserved	
		***Slow frozen/thawed (SFO)***	***Vitrified/warmed (VO)***
N° of vacuoles/100 μm^2^ (vacuole diameter ≥1 μm)	0.40 ± 0.12^a^	2.09 ± 0.85^b^	0.85 ± 0.37^c^

Values are expressed as mean ± SD. Statistical analysis is calculated between columns. Differences in values were considered significant if P <0.05.

^a,b^P <0.0001; ^a,c^P = 0.0005; ^b,c^P <0.0001.

### Cortical granules

**Figure 4:**
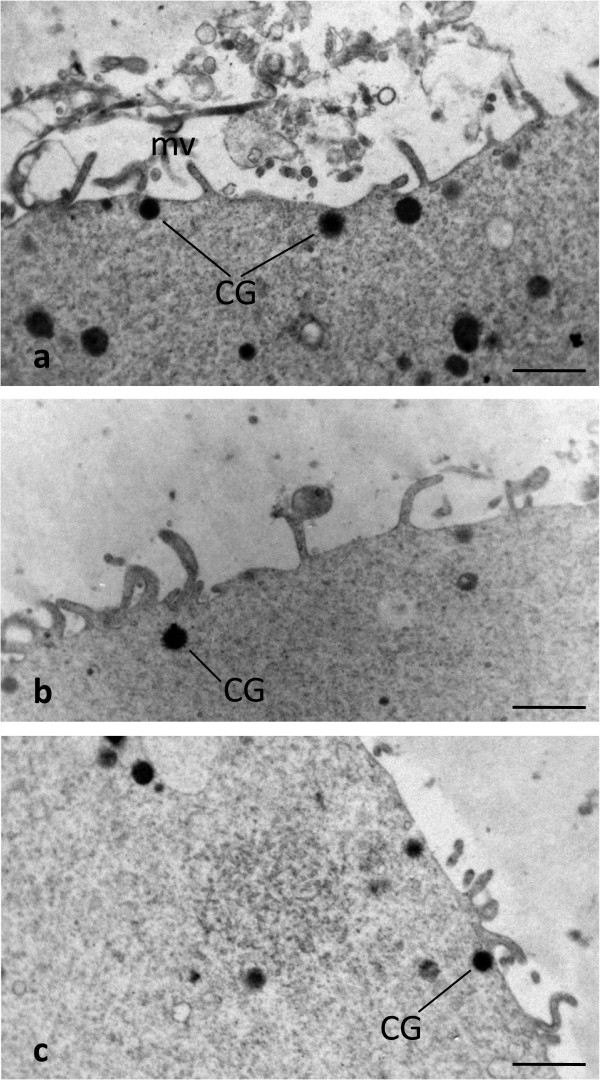
**Human CO, SFO and VO at MII stage.** CG distribution. By TEM, a rim of CG is seen just beneath the oolemma of a CO **(a)**. CG appear instead sparse or isolated forming a discontinuous layer in both SFO **(b)** and VO **(c)**. mv: oocyte microvilli. Bar is: 1 μm **(a-c)**.

In fresh oocytes (CO) the cortex was rich in CG aligned in a continuous array, at the periphery of oocyte, under the oolemma. CG were rounded and showed a diameter varying from 300 to 400 nm (Figure 4a). CG formed instead a discontinuous layer and represented less in both SFO and VO in comparison with CO (Figure 4b and c). The mean number ± SD of CG per 10 μm was 8.10 ± 1.59, 2.95 ± 0.96, and 2.40 ± 0.84 in CO, SFO and VO, respectively. These differences between fresh and cryopreserved groups were highly significant (CO and SFO: P < 0.0001; CO and VO: P < 0.0001). On the contrary, we did not find any significant difference in amount of CG between SFO and VO (P = 0.086) (Table 2).

**Table 2:** **Morphometric evaluation of the amount of CG in human MII oocytes subjected to slow freezing and vitrification with a closed device**

	Fresh controls (CO)	Cryopreserved	
		***Slow frozen/thawed (SFO)***	***Vitrified/warmed (VO)***
N° of CG/10 μm	8.10 ± 1.59^a^	2.95 ± 0.96^b^	2.40 ± 0.84^c^

Values are expressed as mean ± SD. Statistical analysis is calculated between columns. Differences in values were considered significant if P <0.05.

^a,b^P <0.0001; ^a,c^P <0.0001; ^b,c^ P = 0.086*.

*not significant.

## Discussion

Despite over the last 10 years several satisfactory results have been published, yet there is not a defined, generally accepted approach that guarantees a safe routine application of cryopreservation.

As above introduced, the two main protocols applied in the literature are the slow freezing and the vitrification (with open or closed systems) [1].

The cryobiology of slow freezing was based on a slow cooling/rapid thawing method that had already been applied successfully for embryo cryopreservation. All the improvement concerning this technique were mainly focused on the cryoprotectant choice and concentration or the exposure time. The freezing curve has not been changed since the first one designed by Lassalle for embryo freezing [29]. The most significant difference in survival and clinical results seems to be the amount of sucrose used during the freezing and thawing. In detail, a low concentration of sucrose (0.1 mol/L) led to poor survival rates (around 40%) [31]; instead, increasing the amount of sucrose up to 0.3 mol/L significantly improved post-thaw survival rates but implantation and pregnancy rates were low [28, 32]. Bianchi et al. [11, 33] proposed a modified protocol that used a thawing solution in which sucrose concentration was higher than in the freezing solution (0.3 mol/L and 0.2 mol/L respectively). The application of this protocol yielded to better outcome rates (survival rates around 70-80% with a 20% pregnancy rate) in comparison with those obtainable with the above described protocols. This improvement is probably related to the more adequate dehydration achieved with 0.2 mol/L sucrose in the freezing solution compared to 0.1 mol/L or 0.3 mol/L. In addition, the higher (0.3 mol/L) sucrose concentration used during thawing leads to a better controlled water exchange between the inside and outside of the cell stabilizing the membrane and thus avoiding shrinkage.

Conversely, even though the first report of vitrification in embryology was back in 1985 [34], the general application of vitrification in assisted reproduction has been rather limited until recently. Numerous recent publications have shown outstanding results for survival and clinical outcomes using vitrification compared to slow cooling [35–37]. Vitrification methods have been modified over the years to optimize results in humans, by using minimal volumes and very rapid cooling rates, allowing for lower concentrations of cryoprotectants to reduce injuries related to chemical toxicity, osmotic shock, chilling sensitivity and ice nucleation [38, 39]. The use of open carrier devices for vitrification can make a difference on survival rates but sometime they cannot be used due to possible contamination issues. In the open system, in fact, the specimen is in direct contact with liquid nitrogen and this may cause cross contamination even though very unlikely. The closed system is considered safer [17, 18, 40], but slows down the cooling temperature and can reduce the survival rate after thawing, although the replacement of the open vitrification system with a closed system has been reported to have no impact on fertilization, implantation and clinical pregnancy rates [18, 41]. European Union directives on tissue manipulation have been issued by the European Parliament to increase the safety and quality of tissues—including reproductive cells—processed for human re-implantation through the control of equipment, devices, and environment. In these directives, there are no specific indications against direct contact between tissue/cells and liquid nitrogen. Therefore, both open and closed systems comply with European Union directives, as long as aseptic procedures during vitrification-cryostorage-warming are established [16].

In the present study, we evaluated and compared the structural and ultrastructural morphology of human fresh and cryopreserved MII oocytes. Cryopreservation was performed by slow freezing, with PrOH and sucrose (0.2 mol/L in the freezing solution and 0.3 mol/L in the thawing solution), and by vitrification, using a closed device.

### Main characteristics

All CO, SFO and VO appeared normal in shape, dimensions and texture of the ooplasm under LM and low magnification TEM examination. This features are superimposable to those described in our previous reports on human mature oocytes subjected to different protocols of slow cooling [21, 22, 25] or vitrification [23, 42], reinforcing the concept that current cryopreservation protocols, including slow freezing and cryopreservation with a closed device, do not produce major alterations in the oocyte microdomains.

In our study, when observable in sections laying on appropriate planes, the MII spindle appeared regularly shaped and properly positioned not only in the ooplasm of CO but also in that of SFO and VO, confirming that meiotic spindle alterations eventually occurring during cryostorage (both slow freezing and vitrification) can be reversed on rewarming [43]. However, due to specific technical requirements and to the actual difficulty to fully visualize the spindle scaffolding and associated chromosomes in TEM ultrathin sections, we consider other morphological ultrastructural tools, such as confocal laser microscopy, more suitable than TEM for studying spindle morphodynamics during cryopreservation [21].

The finding in both SFO and VO of a good preservation of mitochondrial structure, as in our previous studies [21–23, 25, 42] further emphasizes that both slow freezing and vitrification with a closed device do not produce marked and diffuse alterations in the oocyte microstructure. This does not confirm what reported by other Authors, who described alterations in mitochondrial matrix and cristae in a percentage of oocytes cryopreserved with both slow cooling and vitrification [19, 44]. Due to the many differences among protocols and technique of sampling, it is not possible to hypothesize here an explanation, but this aspect deserves further evaluation.

### Mitochondria-smooth endoplasmic reticulum aggregates and mitochondria-vesicle complexes

We found that M-SER aggregates and small MV complexes were the most common organelles found in all the oocytes cultured for 3–4 hours, both fresh and cryopreserved, with either slow freezing or vitrification.

Small MV complexes, isolated SER tubules and/or SER networks are present in human immature, GV-stage oocytes [42, 45]. Only mature, MII oocytes show fully organized M-SER aggregates of various sizes. These aggregates, according to the biochemical needs of the mature, healthy oocyte, may regulate calcium levels and mitochondrial ATP production, thus potentially contributing to the modulation of calcium-dependent signal transduction pathways at fertilization [46]. M-SER aggregates may have a role in production of substances useful at fertilization and/or in rapid neoformation of nuclear and cytoplasmic membranes during early embryogenesis [47–49].

We did not find evident qualitative differences in the fine structural morphology of M-SER aggregates and MV complexes in both SFO and VO, when compared to CO, after 3–4 hours of culture. In other reports, a percentage of human oocytes, vitrified with open devices, showed small and slender M-SER aggregates [23]. Differently, previous studies on oocytes subjected to slow freezing and treated with PrOH did not evidence any ultrastructural change of these aggregates in respect to fresh controls [21, 25].

In the present study, we also considered the amount of M-SER aggregates, evidencing that they were partially replaced by larger MV complexes in the oocytes maintained in culture for a prolonged period of time (8–9 hours), either SFO or VO. This particular feature, led us to hypothesize that prolonged culture may induce an intracellular membrane “recycling”, causing a discrete enlargement of the MV complexes to which also contribute a considerable SER membrane reassembly. The presence of numerous and large MV complexes was previously described in MII oocytes kept in vitro for 24 (oocytes aged in vitro [50]) or 48 hours (oocytes insemined but not fertilized [47]). Large MV complexes are also present in GV oocytes that have reached MII stage after 24 hour-culture (in vitro matured oocytes [27]). Therefore, changes of MV complexes and SER do not seem related only to the maturative stage of the oocyte at the beginning of the culture, but mainly to the culture period. In the present study, we originally demonstrated that abnormally large MV complexes may form very early during culture, being already present 8–9 hours after oocyte retrieval. The role of these structures is still unknown. We may speculate that they may be associated to impairment of calcium homeostasis, thus reducing the oocyte competence for fertilization after a prolonged culture.

### Vacuoles

In our study, a slight to moderate vacuolization was present in the cytoplasm of SFO. Only a slight vacuolization was instead present in VO. Vacuoles were almost completely absent in CO.

Vacuolization is an important dysmorphism constantly detected in human mature oocytes treated with different protocols of slow freezing [21, 22, 25, 44, 51]. This is explainable as a non-specific response of the oocyte to thermal, osmotic and/or chemical injuries that may occur during cryopreservation. We observed an association of vacuoles with lysosomes and MVBs that are types of organelles rarely present in human healthy mature oocytes and of degenerative significance [44]. The appearance of vacuoles in human oocytes is likely in relation with a reduced oocyte fertilizability and with an impairment of embryo development [52]. The opinions about the presence and extent of vacuolization in human VO are controversial. In fact, some Authors reported an evident vacuolization in oocytes vitrified with both closed (Cryotip) and open (Cryotop) devices [19, 42, 44]. Vacuolization, however, was more severe when a closed device was applied. This may be due to the Cryotip thermosealing and/or to the longer time for closed-vitrification oocyte discharge into the warming solution [19]. In our previous studies, we instead referred a virtual absence of vacuolization in oocytes vitrified using different open devices (Cryoleaf and Cryoloop) [23]. Taken together, all these data suggest the occurrence of a possible cryodevice-dependant vacuolization, that may be also associated to the skill of the operator and even to the sample processing for EM. The different morphometric approaches in vacuole counting and measuring should be also taken into consideration [42]. Our results on SFO well correlate with the data of the recent literature on oocyte cryopreservation, confirming the association between slow freezing and occurrence of vacuolization in the oocyte. We also found only a slight vacuolization in all the VO, significantly lesser than that observed in SFO. Thus our morphometric results, differently from previous qualitative observations, failed in demonstrating a severe incidence of vacuolization in oocytes vitrified with a closed device.

### Cortical granules

We found that amount and density of CG appeared abnormally reduced in both SFO and VO, in comparison with CO.

Usually, CG are regularly present, stratified in one to three rows, in the subplasmalemmal areas of mature oocytes of most mammalian species including humans [47]. They are Golgi-derived, membrane-bounded organelles formed during the early stages of oocyte growth and contain glycosaminoglycans, proteases, acid phosphatases and peroxidases [53]. At fertilization, CG content is suddenly and massively exocytosed by the activated oocyte in the PVS (‘cortical reaction’), leading to a hardening of the inner face of the ZP (‘zona reaction’) and to a consequent inhibition of eventual penetration of supernumerary spermatozoa into the oocyte (polyspermy) [54, 55]. Calcium transients at fertilization seem involved in triggering CG fusion with the oolemma, resulting in the release of their contents into the PVS [56]. Our findings, are comparable to previous studies on slow freezing [21, 22, 25, 44, 51] and vitrification [19, 23, 42, 44], and suggest that a precocious oocyte activation may occur during cryopreservation, irrespective of the protocol applied (slow freezing or vitrification, even with a closed device). This activation leads to a premature CG exocytosis and a consequent diffuse hardening of the ZP, thus greatly impairing oocyte competence to fertilization.

## Conclusions

In conclusion, in this study, human MII oocytes fine structure appeared generally tolerant to both slow freezing and vitrification with a closed system, showing fairly good morphological preservation. However, the appearance of large MV complexes in place of M-SER aggregates in the ooplasm just after 8–9 hours of culture suggests to avoiding extension of culture for human MII oocytes even for a limited number of hours. In addition, the presence of numerous vacuoles in SFO and of only scarce vacuoles in VO implies a different sensitivity of these two groups of oocytes to cryodamage and a better preservation of native oocyte structure after vitrification, even if with a closed system. Thus, in our opinion, vitrification protocol – not only based on open systems, as previously reported by our group, but also employing closed devices - seems to be more effective than slow freezing in preserving oocyte microstructural integrity. Finally, our data support the notion that CG are partially lost during oocyte cryopreservation, irrespective of the protocol applied (slow freezing or vitrification), recommending the use of ICSI as the preferred method of insemination for these oocytes.

## Abbreviations

CG: Cortical granule
CO: Control oocytes
DMSO: Dimethylsulphoxide
EG: Ethylene glycol
EM: Electron microscopy
GV: Germinal vesicle
ICSI: Intracytoplasmic sperm injection
LM: Light microscopy
MII: Metaphase II
M-SER aggregates: Mitochondria-smooth endoplasmic reticulum aggregates
MV complexes: Mitochondria-vesicle complexes
mv: Microvilli
MVBs: Multivesicular bodies
PB: Polar body
PCM: Phase contrast microscopy
PrOH: 1,2-propanediol
PVS: Perivitelline space
SD: Standard deviation
SER: Smooth endoplasmic reticulum
SFO: Slow frozen/thawed oocytes
SSS: Synthetic serum supplement
TEM: Transmission electron microscopy
Va: Vacuoles
VO: Vitrified/warmed oocytes
ZP: Zona pellucida.

## References

[1] Practice Committees of American Society for Reproductive Medicine; Society for Assisted Reproductive Technology (2013). Mature oocyte cryopreservation: a guideline. Fertil Steril.

[2] Chen C (1986). Pregnancy after human oocyte cryopreservation. Lancet.

[3] Jackowski S, Leibo SP, Maxur P (1980). Glycerol permeabilities of fertilized and infertilized mouse ova. J Exp Zool.

[4] Leibo SP (1980). Water permeability and its activation energy of fertilized and unfertilized mouse ova. J Membr Biol.

[5] Coticchio G, Bonu MA, Sciajno R, Sereni E, Bianchi V, Borini A (2007). Truths and myths of oocyte sensitivity to controlled rate freezing. Reprod Biomed Online.

[6] Gook DA, Edgar DH (2007). Human oocyte cryopreservation. Hum Reprod Update.

[7] Edgar DH, Gook DA (2012). A critical appraisal of cryopreservation (slow cooling versus vitrification) of human oocytes and embryos. Hum Reprod Update.

[8] Matson PL, Graefling J, Junk SM, Yovich JL, Edirisinghe WR (1997). Cryopreservation of oocytes and embryos: use of a mouse model to investigate effects upon zona hardness and formulate treatment strategies in an in-vitro fertilization programme. Hum Reprod.

[9] Porcu E, Fabbri R, Seracchioli R, Ciotti PM, Magrini O, Flamigni C (1997). Birth of a healthy female after intracytoplasmic sperm injection of cryopreserved human oocytes. Fertil Steril.

[10] Asghar W, El Assal R, Shafiee H, Anchan RM, Demirci U (2014). Preserving human cells for regenerative, reproductive, and transfusion medicine. Biotechnol J.

[11] Bianchi V, Lappi M, Bonu MA, Borini A (2012). Oocyte slow freezing using a 0.2-0.3 M sucrose concentration protocol: is it really the time to trash the cryopreservation machine?. Fertil Steril.

[12] Levi Setti PE, Porcu E, Patrizio P, Vigiliano V, de Luca R, d'Aloja P, Spoletini R, Scaravelli G (2014). Human oocyte cryopreservation with slow freezing versus vitrification. Results from the National Italian Registry data, 2007–2011. Fertil Steril.

[13] Martínez-Burgos M, Herrero L, Megías D, Salvanes R, Montoya MC, Cobo AC, Garcia-Velasco JA (2011). Vitrification versus slow freezing of oocytes: effects on morphologic appearance, meiotic spindle configuration, and DNA damage. Fertil Steril.

[14] Arav A, Natan Y (2013). Vitrification of oocytes: from basic science to clinical application. Adv Exp Med Biol.

[15] Paffoni A, Guarneri C, Ferrari S, Restelli L, Nicolosi AE, Scarduelli C, Ragni G (2011). Effects of two vitrification protocols on the developmental potential of human mature oocytes. Reprod Biomed Online.

[16] Parmegiani L, Cognigni GE, Filicori M (2012). Vitrification carriers and European regulation. Fertil Steril.

[17] Valbuena D, Póo ME, Aguilar-Gallardo C, Martinez S, Cobo AC, Pellicer A, Simón C (2012). Comparison of Cryotip vs. Cryotop for mouse and human blastomere vitrification. Fertil Steril.

[18] Papatheodorou A, Vanderzwalmen P, Panagiotidis Y, Prapas N, Zikopoulos K, Georgiou I, Prapas Y (2013). Open versus closed oocyte vitrification system: a prospective randomized sibling-oocyte study. Reprod Biomed Online.

[19] Bonetti A, Cervi M, Tomei F, Marchini M, Ortolani F, Manno M (2011). Ultrastructural evaluation of human metaphase II oocytes after vitrification: closed versus open devices. Fertil Steril.

[20] Seet VY, Al-Samerria S, Wong J, Stanger J, Yovich JL, Almahbobi G (2013). Optimising vitrification of human oocytes using multiple cryoprotectants and morphological and functional assessment. Reprod Fertil Dev.

[21] Nottola SA, Macchiarelli G, Coticchio G, Bianchi S, Cecconi S, De Santis L, Scaravelli G, Flamigni C, Borini A (2007). Ultrastructure of human mature oocytes after slow cooling cryopreservation using different sucrose concentrations. Hum Reprod.

[22] Nottola SA, Coticchio G, De Santis L, Macchiarelli G, Maione M, Bianchi S, Iaccarino M, Flamigni C, Borini A (2008). Ultrastructure of human mature oocytes after slow cooling cryopreservation with ethylene glycol. Reprod Biomed Online.

[23] Nottola SA, Coticchio G, Sciajno R, Gambardella A, Maione M, Scaravelli G, Bianchi S, Macchiarelli G, Borini A (2009). Ultrastructural markers of quality in human mature oocytes vitrified using cryoleaf and cryoloop. Reprod Biomed Online.

[24] Camboni A, Martinez-Madrid B, Dolmans MM, Amorim CA, Nottola SA, Donnez J, Van Langendonckt A (2008). Preservation of fertility in young cancer patients: contribution of transmission electron microscopy. Reprod Biomed Online.

[25] Coticchio G, Borini A, Distratis V, Maione M, Scaravelli G, Bianchi V, Macchiarelli G, Nottola SA (2010). Qualitative and morphometric analysis of the ultrastructure of human oocytes cryopreserved by two alternative slow cooling protocols. J Assist Reprod Genet.

[26] Khalili MA, Maione M, Palmerini MG, Bianchi S, Macchiarelli G, Nottola SA (2012). Ultrastructure of human mature oocytes after vitrification. Eur J Histochem.

[27] Shahedi A, Hosseini A, Khalili MA, Norouzian M, Salehi M, Piriaei A, Nottola SA (2013). The effect of vitrification on ultrastructure of human in vitro matured germinal vesicle oocytes. Eur J Obstet Gynecol Reprod Biol.

[28] Borini A, Sciajno R, Bianchi V, Sereni E, Flamigni C, Coticchio G (2006). Clinical outcome of oocyte cryopreservation after slow cooling with a protocol utilizing a high sucrose concentration. Hum Reprod.

[29] Lassalle B, Testart J, Renard JP (1985). Human embryo features that influence the success of cryopreservation with the use of 1,2 propanediol. Fertil Steril.

[30] Rienzi L, Balaban B, Ebner T, Mandelbaum J (2012). The oocyte. Hum Reprod.

[31] Borini A, Bonu MA, Coticchio G, Bianchi V, Cattoli M, Flamigni C (2004). Pregnancies and births after oocyte cryopreservation. Fertil Steril.

[32] Levi Setti PE, Albani E, Novara PV, Cesana A, Morreale G (2006). Cryopreservation of supernumerary oocytes in IVF/ICSI cycles. Hum Reprod.

[33] Bianchi V, Coticchio G, Distratis V, Di Giusto N, Flamigni C, Borini A (2007). Differential sucrose concentration during dehydration (0.2 mol/l) and rehydration (0.3 mol/l) increases the implantation rate of frozen human oocytes. Reprod Biomed Online.

[34] Rall WF, Fahy GM (1985). Ice-free cryopreservation of mouse embryos at -196 degrees C by vitrification. Nature.

[35] Rienzi L, Cobo A, Paffoni A, Scarduelli C, Capalbo A, Vajta G, Remohí J, Ragni G, Ubaldi FM (2012). Consistent and predictable delivery rates after oocyte vitrification: an observational longitudinal cohort multicentric study. Hum Reprod.

[36] Vajta G (2013). Vitrification in human and domestic animal embryology: work in progress. Reprod Fertil Dev.

[37] Potdar N, Gelbaya TA, Nardo LG (2014). Oocyte vitrification in the 21st century and post-warming fertility outcomes: a systematic review and meta-analysis. Reprod Biomed Online.

[38] Kuwayama M, Vajta G, Kato O, Leibo SP (2005). Highly efficient vitrification method for cryopreservation of human oocytes. Reprod Biomed Online.

[39] Vajta G, Nagy ZP (2006). Are programmable freezers still needed in the embryo laboratory? Review on vitrification. Reprod Biomed Online.

[40] Roy TK, Brandi S, Tappe NM, Bradley CK, Vom E, Henderson C, Lewis C, Battista K, Hobbs B, Hobbs S, Syer J, Lanyon SR, Dopheide SM, Peura TT, McArthur SJ, Bowman MC, Stojanov T (2014). Embryo vitrification using a novel semi-automated closed system yields in vitro outcomes equivalent to the manual Cryotop method. Hum Reprod.

[41] Stoop D, De Munck N, Jansen E, Platteau P, Van den Abbeel E, Verheyen G, Devroey P (2012). Clinical validation of a closed vitrification system in an oocyte-donation programme. Reprod Biomed Online.

[42] Palmerini MG, Antinori M, Maione M, Cerusico F, Versaci C, Nottola SA, Macchiarelli G, Khalili MS, Antinori S (2014). Ultrastructure of immature and mature human oocytes after cryotop vitrification. J Reprod Dev.

[43] Boldt J (2011). Current results with slow freezing and vitrification of the human oocyte. Reprod Biomed Online.

[44] Gualtieri R, Mollo V, Barbato V, Fiorentino I, Iaccarino M, Talevi R (2011). Ultrastructure and intracellular calcium response during activation in vitrified and slow-frozen human oocytes. Hum Reprod.

[45] El Shafie M, Sousa M, Windt M-L, Kruger TF (2000). An Atlas of the Ultrastructure of Human Oocytes.

[46] Makabe S, Van Blerkom J, Nottola SA, Naguro T (2006). Atlas of Human Female Reproductive Function. Ovarian Development to Early Embryogenesis after In Vitro Fertilization.

[47] Motta PM, Nottola SA, Micara G, Familiari G (1988). Ultrastructure of human unfertilized oocytes and polyspermic embryos in an IVF-ET program. Ann N Y Acad Sci.

[48] Motta PM, Nottola SA, Makabe S, Heyn R (2000). Mitochondrial morphology in human fetal and adult female germ cells. Hum Reprod.

[49] Motta PM, Nottola SA, Familiari G, Makabe S, Stallone T, Macchiarelli G (2003). Morphodynamics of the follicular-luteal complex during early ovarian development and reproductive life. Int Rev Cytol.

[50] Bianchi S, Macchiarelli G, Micara G, Aragona C, Maione M, Nottola SA (2013). Ultrastructural and morphometric evaluation of aged cumulus-oocyte-complexes [abstract]. It J Anat Embryol.

[51] Gualtieri R, Iaccarino M, Mollo V, Prisco M, Iaccarino S, Talevi R (2009). Slow cooling of human oocytes: ultrastructural injuries and apoptotic status. Fertil Steril.

[52] Ebner T, Moser M, Sommergruber M, Gaiswinkler U, Shebl O, Jesacher K, Tews G (2005). Occurrence and developmental consequences of vacuoles throughout preimplantation development. Fertil Steril.

[53] Familiari G, Heyn R, Relucenti M, Nottola SA, Sathananthan AH (2006). Ultrastructural dynamics of human reproduction, from ovulation to fertilization and early embryo development. Int Rev Cytol.

[54] Sathananthan AH, Selvaraj K, Girijashankar ML, Ganesh V, Selvaraj P, Trounson AO (2006). From oogonia to mature oocytes: inactivation of the maternal centrosome in humans. Microsc Res Tech.

[55] Sathananthan AH (2013). Ultrastructure of human gametes, fertilization and embryos in assisted reproduction: a personal survey. Micron.

[56] Gardner DK, Sheehan CB, Rienzi L, Katz-Jaffe M, Larman MG (2007). Analysis of oocyte physiology to improve cryopreservation procedures. Theriogenology.

